# A case of *Streptococcus canis* bacteremia, osteomyelitis, sacroiliitis, myositis, and abscess

**DOI:** 10.1186/s12879-022-07580-3

**Published:** 2022-07-15

**Authors:** A. L. Van Tol, B. Tang, I. D. Mackie

**Affiliations:** 1 Department of Family Medicine, Queen’s University, Kingston, ON Canada; 2 Division of General Internal Medicine, University of Toronto, Toronto, ON Canada; 3 Division of General Internal Medicine, Vancouver General Hospital and University of British Columbia, Vancouver, BC Canada

**Keywords:** *Streptococcus canis*, Zoonosis, Bacteremia, Sepsis, Osteomyelitis, Abscess, Cellulitis

## Abstract

**Background:**

*Streptococcus canis* is a group G beta-hemolytic *Streptococcus* species which normally resides on the skin and mucosal surfaces of dogs*.* Although it rarely causes infection in humans, our case and review of relevant literature demonstrate that this multi-host pathogen may be responsible for metastatic infection. We present an appropriate management strategy in such cases.

**Case presentation:**

A previously healthy 26-year-old male presented to the emergency department with a 2-day history of erythema, pain, and swelling of the left ankle and foot, consistent with acute cellulitis. The patient was initially discharged home with a plan to complete a course of IV cefazolin as an outpatient, but later recalled after two sets of blood cultures grew gram positive cocci. Blood cultures speciated as *Streptococcus canis.* This was performed by identifying beta hemolytic strep on blood agar, then typed as Lancefield group G, followed by MALDI-TOF which distinguished *S. canis*. History was unremarkable except for a 2-week history of lower back pain precipitated by a wrestling injury. There was no canine bite or scratch wound, although the patient lives with a dog. CT spine was obtained which demonstrated right piriformis myositis and S1 osteomyelitis. MRI additionally demonstrated right erector spinae myositis, right sacroiliitis, and multiple collections in the right posterior paraspinal soft tissues. Transthoracic echocardiogram did not demonstrate valvular vegetations. The *S. canis* isolate was pan-susceptible and the patient was ultimately discharged home and completed a 8-week course of IV penicillin G. After completion of therapy, his symptoms, repeat imaging, and biochemical markers suggested resolution of infection on follow-up.

**Conclusions:**

We suggest that management of *S. canis* bacteremia should involve consideration of screening for metastatic infection and infectious diseases consultation. However, despite its potential for systemic involvement, *S. canis* is often susceptible to narrow spectrum antibiotics, and may be treated with penicillins.

## Key Points

1. *S. canis* does not require a clear portal of entry to cause infection
2. When *S. canis* infection is identified, screening for sites of metastatic infection should be considered
3. *S. canis* infection is typically susceptible to narrow-spectrum antibiotics

## Background

*Streptococcus canis* is a bacterium most commonly found in the normal oral and skin flora of dogs and is well known in veterinary medicine to cause a variety of infections including skin and soft tissue infection, bacteremia, and pneumonia [1]. Although it rarely causes infection in humans, our case and review of relevant literature demonstrate that this bacterium can cause various types of infection, including rare cases of metastatic infection, in otherwise healthy hosts.

## Case presentation

A previously healthy 26-year-old male presented to the emergency department (ED) at Vancouver General Hospital with a 2-day history of erythema, pain, and swelling of his left ankle and foot, consistent with uncomplicated cellulitis. Laboratory investigations and blood cultures were obtained in the ED, and the patient was discharged home with a plan to complete a course of intravenous (IV) cefazolin as an outpatient. He was recalled 17 h later when two sets of blood cultures returned positive for gram positive cocci in chains.

On further inquiry, the patient endorsed a 2-week history of mechanical lower back pain, initially precipitated by a wrestling injury. He endorsed 2/10 pain to the left foot at the location of the cellulitis. Review of systems was otherwise unremarkable. The patient remained afebrile with no constitutional symptoms and no lower extremity weakness. There was no known injury to the foot or ankle prior to presentation.

On physical examination, the patient’s initial vital signs were: temperature 36.4C, heart rate 138 bpm, respiratory rate 18, blood pressure 123/62 mmhg, SpO_2_ 94% on room air. The left foot was diffusely erythematous from the distal foot to proximal ankle. The involved area was non-purulent and blanchable. The foot was tender to palpation, but there was no fluctuance or crepitus. Cardiac, respiratory, abdominal, and neurological exams were unremarkable. There were no cardiac murmurs or stigmata of infective endocarditis. The spine was not assessed at this time.

**Fig. 1:**
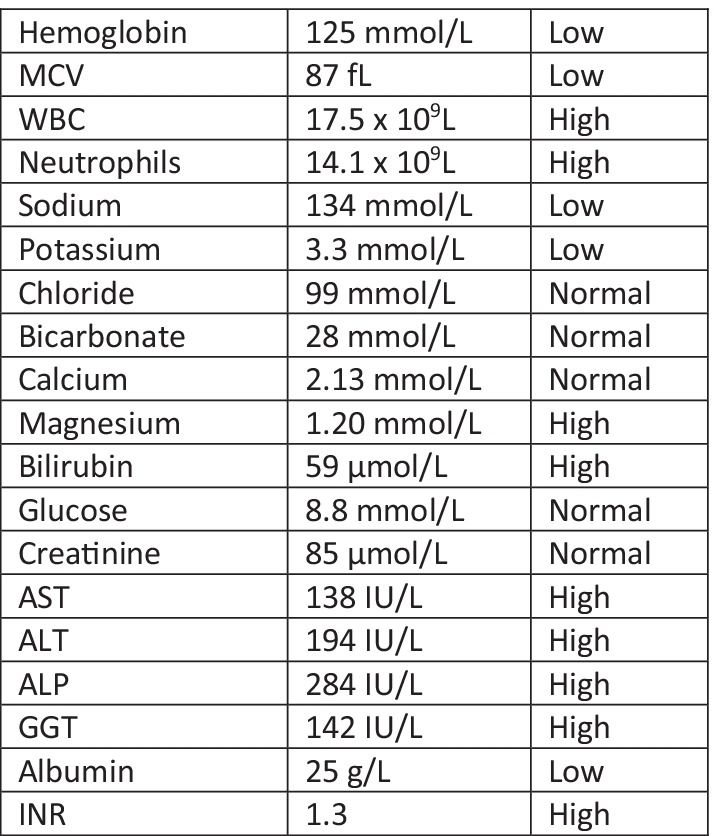
Laboratory values of the patient on admission

Initial laboratory investigations included complete blood count (CBC), electrolytes, extended electrolytes, creatinine, liver function tests (LFTs). These investigations demonstrated anemia, leukocytosis (primarily neutrophilic), mixed hepatocellular and cholestatic elevation of liver enzymes, and abnormal liver function tests (Fig. 1). X-ray imaging demonstrated no evidence of left ankle and foot fracture or osteomyelitis, with no joint effusion on ankle ultrasound.

The clinical presentation was consistent with bacteremia secondary to cellulitis, and the patient received IV fluid resuscitation and empiric treatment with IV vancomycin. After 35 h, two sets of blood cultures in anaerobic and aerobic bottles speciated as *S. canis*, and antibiotics were changed to ampicillin 2 g IV every 4 h (Q4H) per recommendation from our infectious diseases team. Speciation of blood cultures prompted further history taking and the patient endorsed occasional contact with a housemate’s dog, but without history of bite or trauma to the foot.

**Fig. 2:**
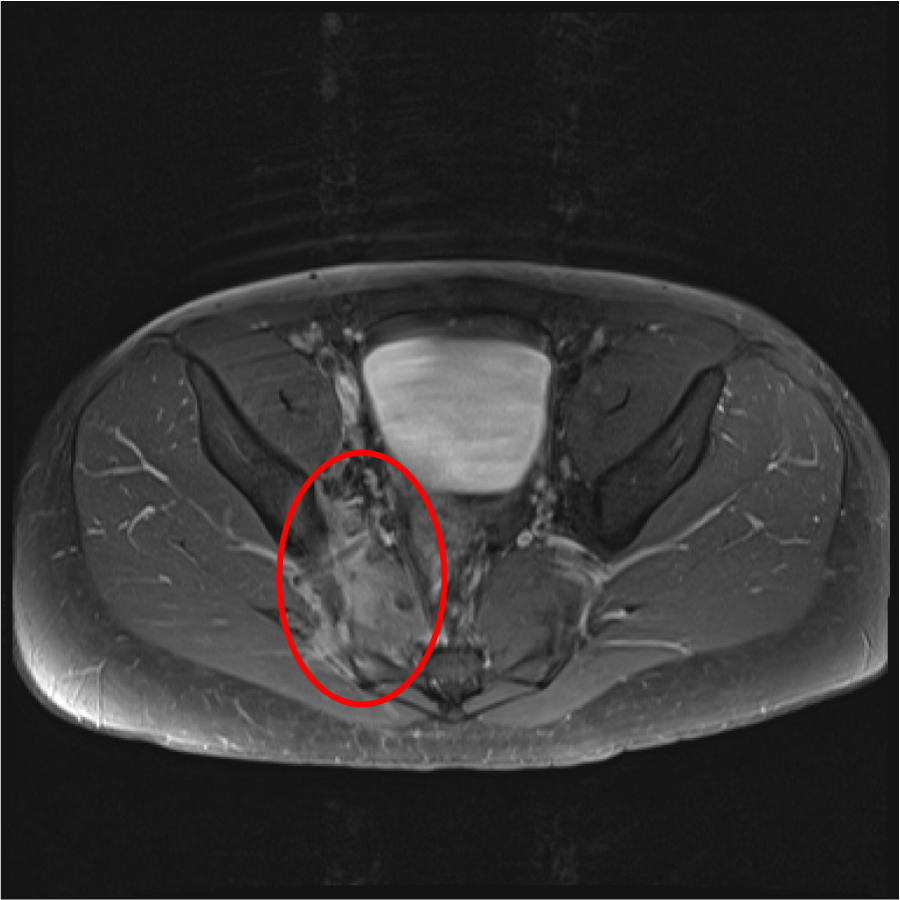
Sagittal T1, sagittal T2, axial T2, with addition of sagittal STIR and resolve sequences. There is increased signal in the right piriformis muscle consistent with myositis (circled)

**Fig. 3:**
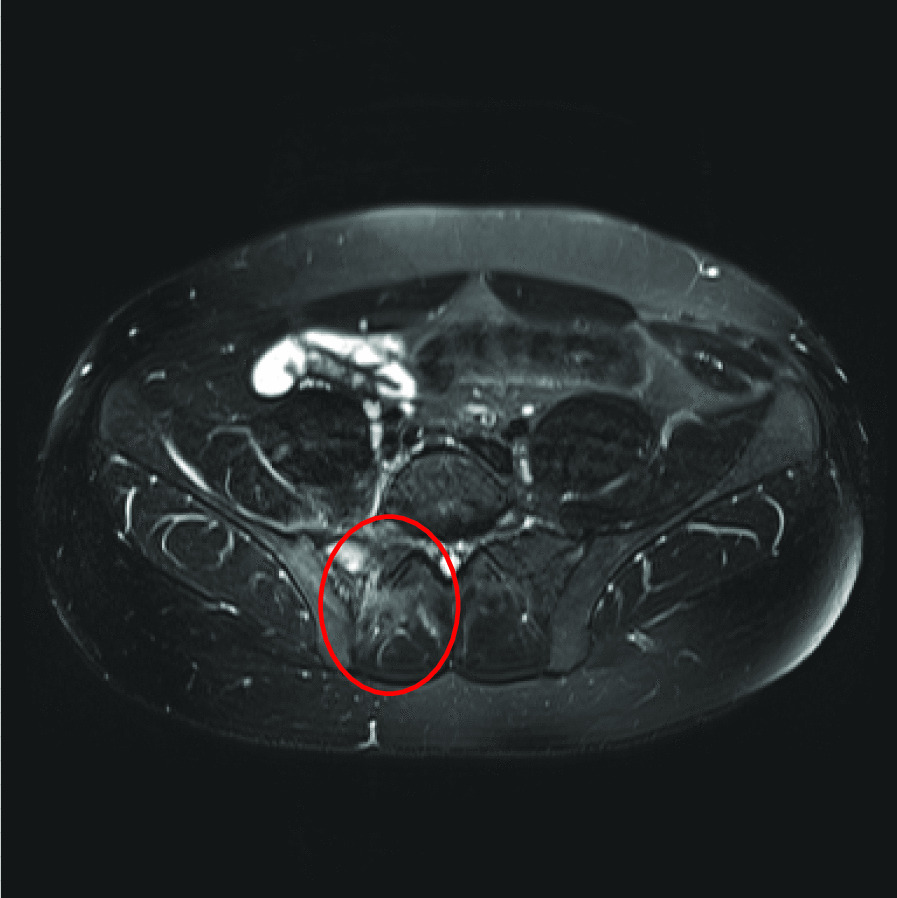
Sagittal T1, sagittal T2, axial T2, with addition of sagittal STIR and resolve sequence. High signal is notes within the inferior aspect of the right erector spinae muscles at the level of the pelvis, suggesting myositis (circled)

**Fig. 4:**
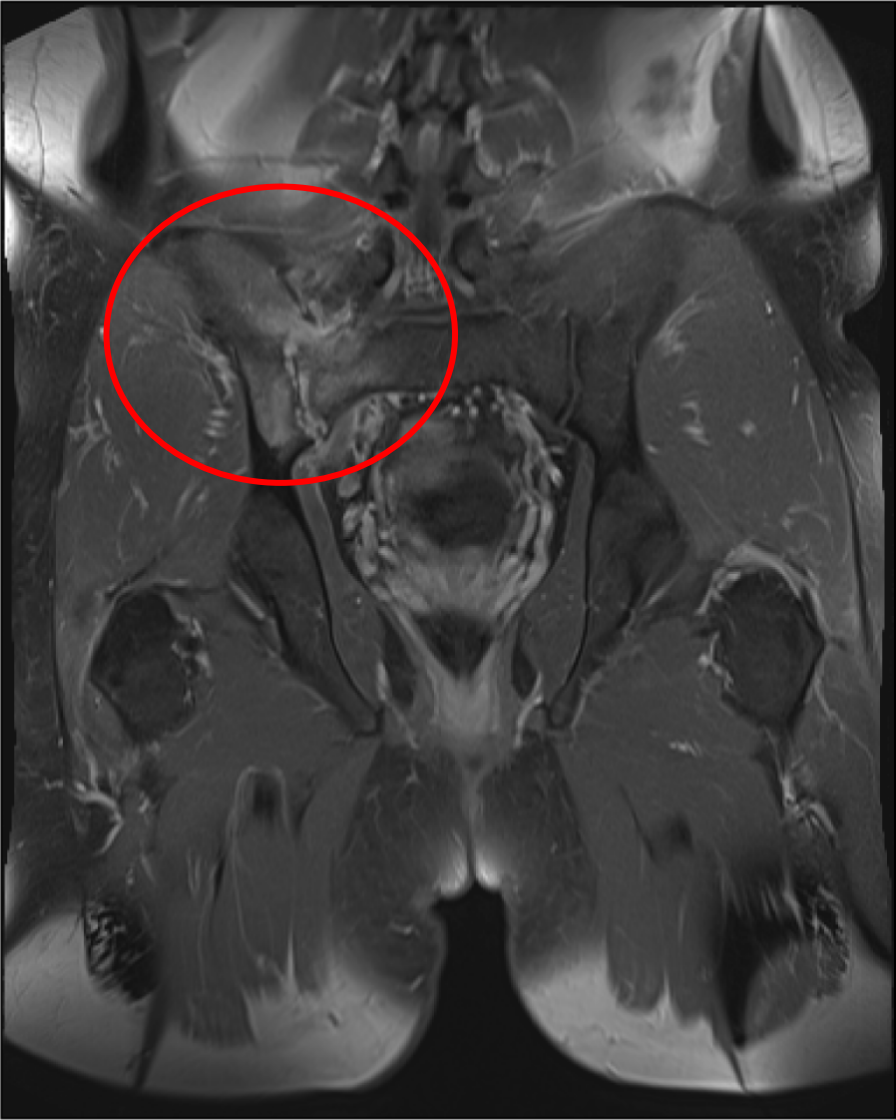
Sagittal T1, sagittal T2, axial T2, with addition of sagittal STIR and resolve sequences. MRI demonstrated small amount of fluid in the right SI joint with associated bone marrow edema suggesting sacroiliitis and osteomyelitis (circled)

Given the patient’s ongoing back pain, which was present through his admission, both computed tomography (CT) and magnetic resonance imaging (MRI) spine were ultimately performed and suggestive of myositis of the right piriformis (Fig. 2) and right erector spinae (Fig. 3), S1 spinal osteomyelitis, and right sacroiliitis (Fig. 4). There were also multiple small fluid collections, the largest measuring 25 × 19 mm, in the right posterior paraspinal soft tissue. These collections were not amenable to drainage per interventional radiology. Transthoracic echocardiogram (TTE) did not demonstrate infective endocarditis. Both liver enzymes and liver function tests rapidly normalized, consistent with resolution of hypoperfusion in the context of sepsis. Abdominal CT did not demonstrate any underlying liver abscess or other pathology.

Ultimately, blood cultures cleared rapidly after 2 days of treatment with IV ampicillin. The patient’s foot pain, redness, and swelling improved over the course of his 1-week hospitalization, and he was discharged home on the same dose of IV penicillin G with plans to complete a 6-week course. This was administered through an outpatient IV pump. An outpatient MRI was ordered to monitor response to therapy, and follow-up was arranged with the outpatient infectious diseases team.

After discharge, the patient’s course of IV penicillin G was extended to 8 weeks in total due to ongoing back pain. He had a repeat MRI (10 weeks after his previous MRI) which demonstrated improving piriformis muscle inflammation, resolution of piriformis fluid collections, although with ongoing SI joint inflammation. His C-reactive protein (CRP) first normalized 12 weeks after initiation of antibiotics and serial CRPs after stopping antibiotics remained normal. At this point, given resolution of his symptoms, improvement of imaging findings, and normalization of CRP, he was discharged from ongoing follow-up.

## Discussion and conclusions

The patient in our case presented with sepsis secondary to *S. canis* bacteremia and was ultimately found to have diffuse metastatic infection including osteomyelitis, sacroiliitis, myositis, and abscess. Metastatic infection may have occurred via hematogenous spread, although this was not confirmed. TTE alone could not rule out the possibility of left-sided infective endocarditis with seeding of infection via septic emboli. After discussion with our infectious diseases team, we rationalized that a transesophageal echocardiogram (TEE) was not indicated as there was a low probability of identifying pathology on TEE that would have changed management. The patient’s osteomyelitis already necessitated at least 6 weeks of antibiotic therapy and there was no evidence of aortic root abscess on either TTE or electrocardiogram (ECG) that would have required surgical management.

Our case demonstrates the importance of considering metastatic infection in cases of *S. canis* bacteremia. Although *S. canis* most frequently causes skin and soft tissue infection, it has been implicated in a variety of other infections including bacteremia, osteoarticular infections, and even pneumonia (Table 1). Infective endocarditis secondary to *S. canis* bacteremia has been reported in at least three previous cases [3–5]. However, the risk of endocarditis given the finding of a positive blood culture is not known [6]. The presence of endocarditis in this case likely would not have changed management given concomitant osteomyelitis. However, it may be an important consideration in other causes of metastatic infection due to *S. canis* bacteremia.

**Table 1:** Frequency of clinical manifestations associated with *S. canis* infection in humans

Diagnosis	Frequency (N)*	References
Skin-soft tissue infection	39	[2, 7, 10, 11, 13, 15]
Bacteremia	17	[2, 6, 9, 12, 16, 17]
Endocarditis	3	[3–5]
Urinary tract infection	3	[2]
Osteoarticular infection	4	[2, 11, 14]
Nosocomial pneumonia	1	[2]
Central nervous system infection	1	[2]

*We searched PubMed for the following search terms: *Streptococcus canis* AND (Bacteremia OR endocarditis OR osteomyelitis) with no date restrictions, yielding 19 studies of interest. These included both case studies and epidemiological studies

Another interesting characteristic of this case was that there was no clear portal of entry for infection. This patient did have a recent wrestling injury to his back which resulted in mechanical back pain, however, there was no known break in the skin, dog bite, or scratch injury. It remains unknown how the patient was infected with *S. canis.* We hypothesize that the patient’s skin may have been colonized by *S. canis* through interaction with his housemate’s dog, however, there was insufficient evidence to confirm this. In fact, other cases of *S. canis* infection also reported no clear portal of entry, including two cases which resulted in the development of bacteremia [7, 8] and one case of infective endocarditis [3]. Fortunately, when infection does occur, most cases of *S. canis* infection are susceptible to narrow spectrum antibiotics such as penicillin (Table 2).

**Table 2:** Antibiotic susceptibilities of *S. canis* in humans

Antibiotic	Susceptible (N)	Resistant (N)	References
Tetracycline	64 (72%)	24	[2, 14]
Erythromycin	76 (86%)	12	[2, 4, 7,13, 14, 16, 17]
Penicillin	83 (95%)	4	[2–4, 7, 13, 14, 16, 17]
Gentamycin	85 (97%)	3	[2, 3, 12]
Clindamycin	4 (100%)	0	[4, 13, 14, 16, 17]
Vancomycin	3 (100%)	0	[4, 13, 16]
Amoxicillin	2 (100%)	0	[12, 16, 17]
Cefaltoin	2 (100%)	0	[4, 16, 17]
Cefotaxime	2 (100%)	0	[7, 14]
Ceftriaxone	2 (100%)	0	[7, 14]

In summary, our recommendations for the management of *S. canis* bacteremia include consideration of screening for endocarditis and to consider the possibility for metastatic infection, per the patient’s presentation. We also recommend consultation with an infectious disease specialist, given the rarity of this bacterium, and treatment with narrow spectrum antibiotics when susceptibilities are available.

## Patient perspective

The patient was consulted during the information gathering process to describe their symptoms and experiences. A copy of the manuscript will be forwarded to the patient on completion.

## Data Availability

Data sharing is not applicable to this article as no datasets were generated or analysed during the current study.

## Abbreviations

IV: Intravenous
Q4H: Every 4 h
ED: Emergency department
CBC: Complete blood count
LFTs: Liver function tests
CT: Computed tomography
MRI: Magnetic resonance imaging
TTE: Transthoracic echocardiogram
TEE: Transesophageal echocardiogram
ECG: Electrocardiogram
CRP: C-reactive protein
